# Adjuvant Therapy of High-Risk (Stages IIC–IV) Malignant Melanoma in the Post Interferon-Alpha Era: A Systematic Review and Meta-Analysis 

**DOI:** 10.3389/fonc.2020.637161

**Published:** 2021-02-18

**Authors:** Konstantinos Christofyllakis, Claudia Pföhler, Moritz Bewarder, Cornelia S. L. Müller, Lorenz Thurner, Torben Rixecker, Thomas Vogt, Stephan Stilgenbauer, Krista Yordanova, Dominic Kaddu-Mulindwa

**Affiliations:** ^1^ Department of Hematology, Oncology, Clinical Immunology and Rheumatology, Medical School, University of Saarland, Homburg, Germany; ^2^ Department of Dermatology, Venerology and Allergology, Medical School, University of Saarland, Homburg, Germany

**Keywords:** melanoma, adjuvant, immunotherapy, BRAF mutation, meta-analysis, checkpoint inhibitors, BRAF/ MEK inhibitors

## Abstract

**Introduction:**

Multiple agents are approved in the adjuvant setting of completely resected high-risk (stages IIC–IV) malignant melanoma. Subgroups may benefit differently depending on the agent used. We performed a systematic review and meta-analysis to evaluate the efficiency and tolerability of available options in the post interferon era across following subgroups: patient age, stage, ulceration status, lymph node involvement, BRAF status.

**Methods:**

The PubMed and Cochrane Library databases were searched without restriction in year of publication in June and September 2020. Data were extracted according to the PRISMA Guidelines from two authors independently and were pooled according to the random-effects model. The predefined primary outcome was recurrence-free survival (RFS). Post-data extraction it was noted that one trial (BRIM8) reported disease-free survival which was defined in the exact same way as RFS.

**Results:**

Five prospective randomized placebo-controlled trials were included in the meta-analysis. The drug regimens included ipilimumab, pembrolizumab, nivolumab, nivolumab/ipilimumab, vemurafenib, and dabrafenib/trametinib. Adjuvant treatment was associated with a higher RFS than placebo (HR 0.57; 95% CI= 0.45–0.71). Nivolumab/ipilimumab in stage IV malignant melanoma was associated with the highest RFS benefit (HR 0.23; 97.5% CI= 0.12–0.45), followed by dabrafenib/trametinib in stage III BRAF-mutant melanoma (HR 0.49; 95% CI= 0.40–0.59). The presence of a BRAF mutation was associated with higher RFS rates (HR 0.30; 95% CI= 0.11–0.78) compared to the wildtype group (HR 0.60; 95% CI= 0.44–0.81). Patient age did not influence outcomes (≥65: HR 0.50; 95% CI= 0.36–0.70, <65: HR 0.58; 95% CI= 0.46–0.75). Immune checkpoint inhibitor monotherapy was associated with lower RFS in non-ulcerated melanoma. Patients with stage IIIA benefited equally from adjuvant treatment as those with stage IIIB/C. Nivolumab/ipilimumab and ipilimumab monotherapy were associated with higher toxicity.

**Conclusion:**

Adjuvant therapy should not be withheld on account of advanced age or stage IIIA alone. The presence of a BRAF mutation is prognostically favorable in terms of RFS. BRAF/MEK inhibitors should be preferred in the adjuvant treatment of BRAF-mutant non-ulcerated melanoma.

## Introduction

The incidence of malignant melanoma (MM) increases consistently (39% between 2006 and 2016) with a current incidence of over 132.000 estimated cases worldwide each year (1, 2). Low risk MM (stages I-IIB) can be effectively treated with surgical excision only (3, 4). In contrast, high-risk MM (stages IIC-IV) with no evidence of disease (NED) after excision is associated with a worse survival rate (5) and therefore an efficient and tolerable adjuvant therapy is needed (4, 6). Interferon alpha (IFN-α) has lost its relevance in the wake of new therapeutic options due to its inconsistent impact on overall survival (OS) and high toxicity (7–9). After IFN-α, ipilimumab was the first agent to be approved for stage III (10). However, due to its unfavorable side effect profile, it was soon replaced by nivolumab and pembrolizumab (11). A recent phase II trial demonstrated the superior efficacy of the nivolumab/ipilimumab combination versus nivolumab or placebo in stage IV MM with NED (12). For patients with *BRAF-*V600 mutant (BRAF*mut*) MM, targeted adjuvant therapy is another option. While initial results with the BRAFi vemurafenib were not encouraging, the combination of the BRAFi dabrafenib with the MEKi trametinib demonstrated a clear benefit versus placebo in stage III disease (13, 14). Despite these significant developments, there is still no standard of care for the adjuvant therapy of high-risk MM, especially in the presence of a BRAF driver mutation (15).

Previous meta-analyses of adjuvant therapy for MM either included IFN-α, did not include subgroup-specific data or lacked a direct comparison of nivolumab versus placebo within a randomized placebo-controlled trials (RCT) (8, 16).

## Materials and Methods

### Objective

This systematic review and meta-analysis aimed to compare the efficacy of modern agents in the adjuvant setting of cutaneous MM versus placebo with specific regard to different subgroups [patient age, stage, primary tumor ulceration, number of involved lymph nodes (LN), type of LN involvement (micro- or macrometastases) and BRAF mutational status]. Methodology and reporting follow the PRISMA guidelines (Preferred Reporting Items for Systematic Reviews and Meta-Analysis) (17), a checklist is provided in**eTable 1, supplement**. The meta-analysis is registered on the Open Science Framework (Registration DOI: 10.17605/OSF.IO/SGPHN, protocol accessible on: https://osf.io/m9vr5)

### Data Sources, Search Strategy, and Data Extraction

The PubMed and Cochrane Library databases were searched in June 2020 using the terms “melanoma” AND “adjuvant” and the filter “clinical trial”. An updated search was performed on September 14^th^, 2020 (search strategy in **eFigure 1, supplement**). RCT (phase 2 or 3) comparing adjuvant treatment with placebo or an FDA- or EMA approved agent in patients with MM with NED published in English were included. We excluded systematic reviews, meta-analyses, abstracts, trials including neoadjuvant treatment, IFN-α as well as non-placebo-controlled RCT. Baseline participant demographics and outcome data were extracted including: type and name of the trial, primary outcome for the whole population, and separately for the following subgroups: patients <65 and ≥65 years of age, ulceration status, number of positive LN, presence of micro- or macrometastases, stage and BRAF status. Two authors (KC and KY) conducted the systematic review and data extraction independently. Conflicts were resolved by a third author (DK-M).

### Comparators and Data Analysis

We conducted a meta-analysis of the summary statistic hazard ratio (HR) with corresponding 95% confidence intervals (CI) for each trial. Data from each trial were pooled using the random effects (DerSimonian-Laird) model. Statistical heterogeneity between the trials was assessed using Cochran´s Q test and I². All statistical analyses were conducted using StatsDirect version 3.3.0. Results were presented with forest plots. Two-sided *P < *0.05 was deemed statistically significant.

### Risk of Bias

Trial quality and risk of bias on study level were assessed using the revised Cochrane tool for assessing risk of bias in randomized trials (RoB2 tool) (18) by two authors independently (KC and KY). Conflicts were referred to a third author (DK-M). A potential presence of publication bias was assessed visually with funnel plots and formally using Egger’s regression asymmetry test (19).

## Results

### Study Selection

**Figure 1:**
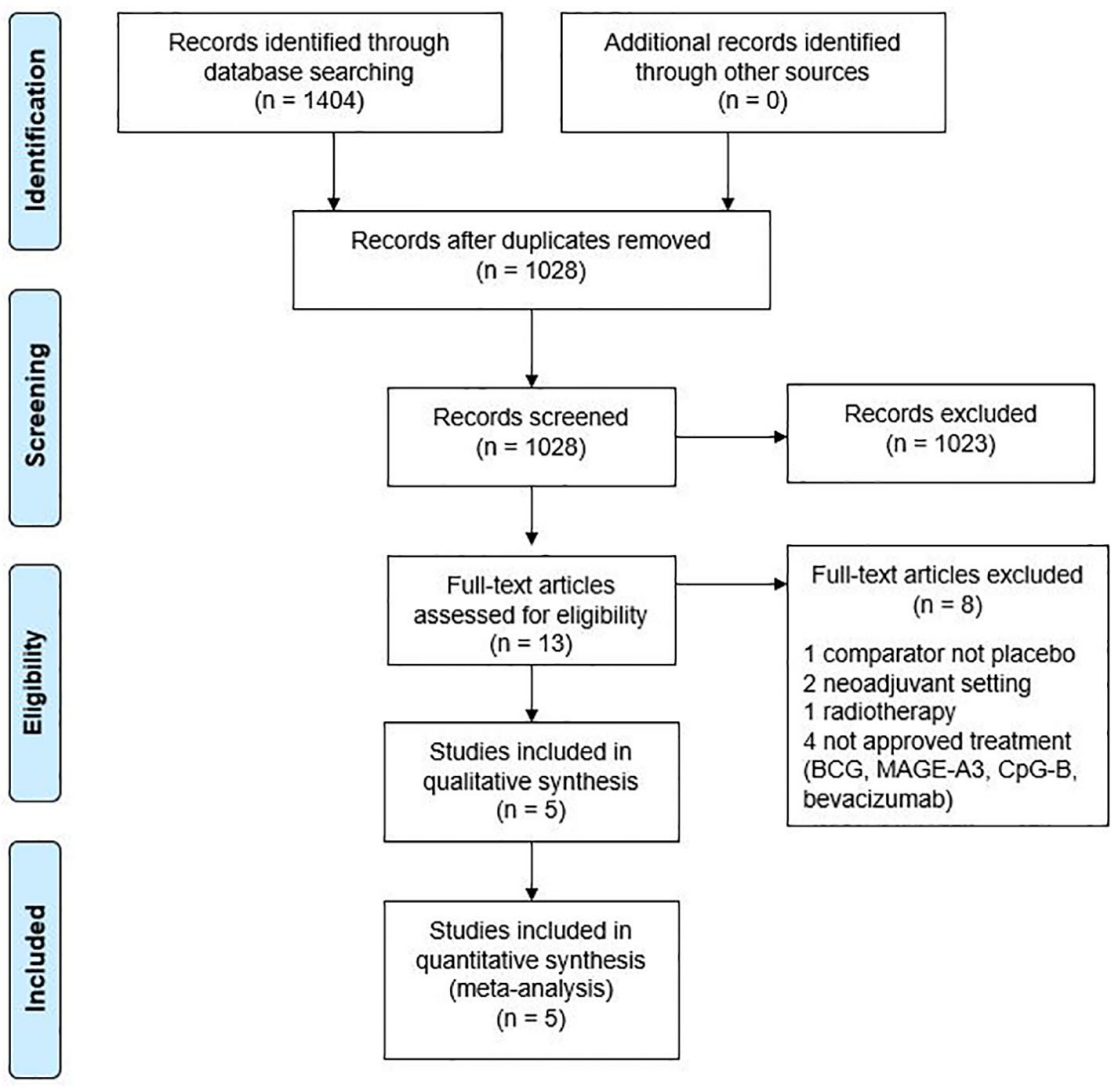
Meta-analysis flowchart. BCG, bacillus Calmette-Guerin; MAGE-A3, melanoma antigen A3.

We identified 1,404 studies in total. After assessment for eligibility, five randomized, double blind, placebo-controlled trials were included in the meta-analysis. A flowchart is provided in **Figure 1** and an overview of these trials in **Table 1**.

**Table 1:** Overview of the characteristics of the included studies.

Trial	Comparison	Randomised patients (n)	Dose schedule	Duration of treatment	Median follow up	Primary endpoint HR, (95% CI)
**EORC-18071**	Ipilimumab versus placebo	951	10 mg/kg i.v. q3w for four doses, then every 3 months for 3 years	3 years	2.74 years	RFS, 0.76 (0.64–0.89)
**COMBI-AD**	Dabrafenib plus Trametinib versus placebo	870	Dabrafenib 150 mg 2× day + trametinib 2 mg 1× day	1 year	2.9 years	RFS, 0.49 (0.40–0.59)
**BRIM8**	Vemurafenib versus placebo	Cohort 1: 314	Vemurafenib tablets (960 mg 2× day for 52 weeks [13 × 28-day cycles])	52 weeks	33.5 months	DFS, 0.55 (0.38–0.80)
		Cohort 2: 184	as Cohort 1	52 weeks	30.8 months	DFS, 0.81 (0.55–1.19)
**EORTC-1325**	Pembrolizumab versus placebo	1019	200 mg i.v. q3w for a total of 18 doses	Approximately 1 year	15 months	RFS, 0.57 (0.43–0.74)
**IMMUNED**	Nivolumab versus placebo	167	3 mg/kg nivolumab q3w	Up to 1 year	28.4 months	RFS, 0.56 (0.33–0.94)
	Nivolumab plus Ipilimumab versus placebo		1 mg/kg i.v. nivolumab q3w plus 3 mg/kg i.v. ipilimumab q3w for four doses, followed by 3 mg/kg i.v. nivolumab q2w			RFS, 0.23 (0.12–0.45)*

* 97.5% CI.

### Study Characteristics

The following drug regimens were compared versus placebo: pembrolizumab (EORTC-1325), ipilimumab (EORTC-18071), vemurafenib (BRIM8), nivolumab/ipilimumab, nivolumab (IMMUNED) and dabrafenib/trametinib (COMBI-AD). The BRIM8 trial incorporated two cohorts of patients based on the tumor stage: cohort 1 (IIC-IIIB) and cohort 2 (IIIC). In total, data from 3505 patients were evaluated. For the EORTC-18071 and COMBI-AD trials, updated data published in 2016 and 2018 were used, respectively. Staging was performed according to the 7^th^ edition of the American Joint Committee on Cancer (AJCC) in the COMBI-AD, EORTC-1325 and BRIM8 trials. The EORTC-18071 trial included only patients with stage III disease according to the 6^th^ AJCC edition. However, there are no differences in stage III definition between 6^th^ and 7^th^ edition. Only nodal micrometastatic disease size > 1 mm was included in the EORTC-1325, EORTC-18071, BRIM8, and COMBI-AD trials. The IMMUNED trial included only patients with stage IV with NED, whose distinction from stage III does not differ between the 7^th^ and 8^th^ editions. Thus, cross-trial comparability is warranted. Taken together, all trials enrolled patients with completely resected stage IIC to IV cutaneous MM. The median follow-up of the studies ranged from 15 months to 2.9 years. The primary endpoint of three of the studies was RFS defined as the time from randomization to disease recurrence or death. The BRIM8 trial used disease-free survival (DFS) as the primary endpoint defined as the time from randomization until the date of the first disease recurrence or death. As those two definitions are identical, RFS will be used from now on for purposes of simplicity. All trials except BRIM8 met their primary endpoint. All patients included in the COMBI-AD and BRIM8 trials had BRAF*mut* melanoma. In the EORTC-1325 and IMMUNED trials 49% and 45% of patients respectively had BRAF*mut* MM. The EORC-18071 did not report BRAF mutational status (**eTable 2, supplement**).

### Recurrence-Free Survival

**Figure 2:**
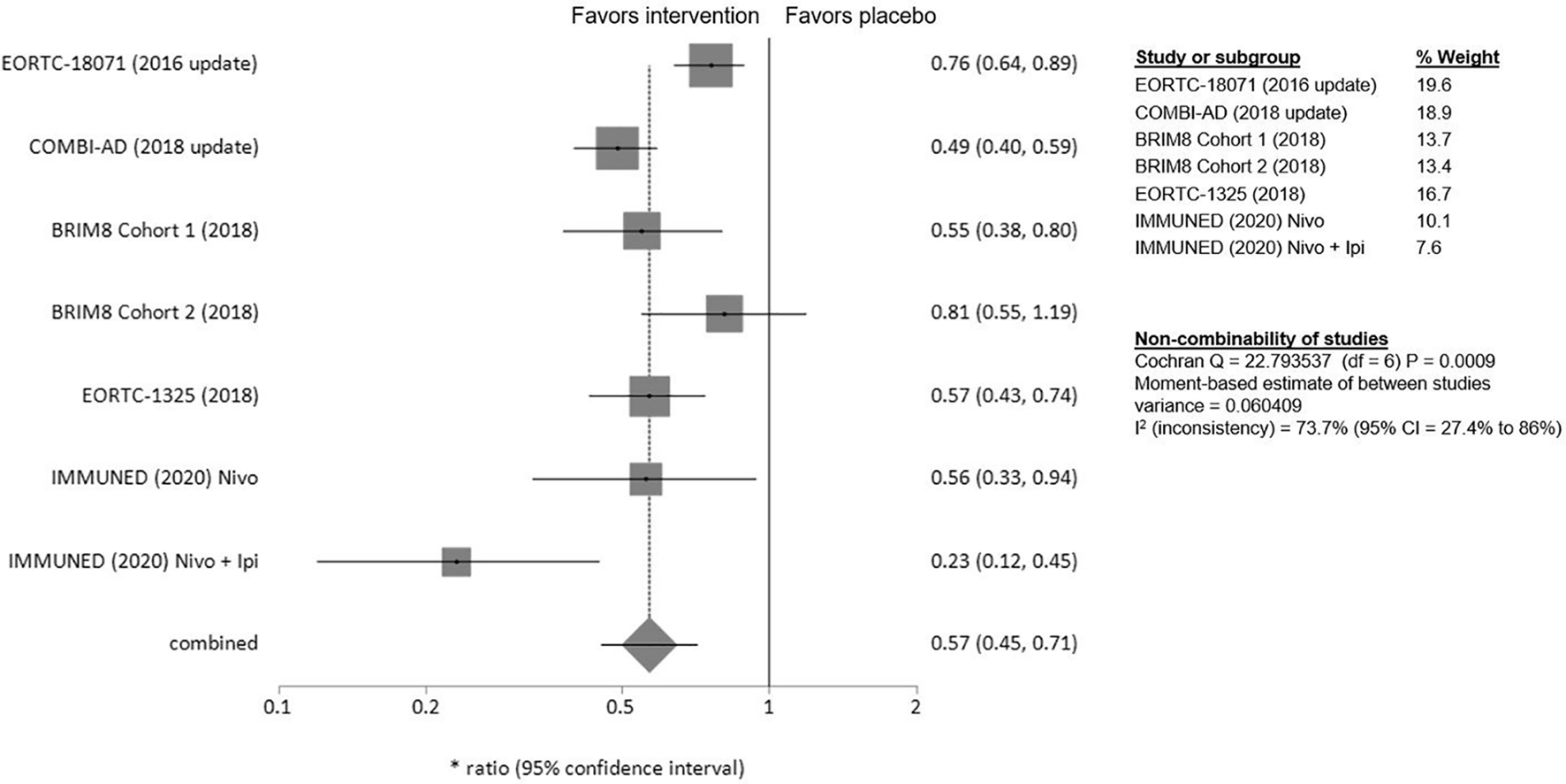
Forest plot for primary outcome analysis on relapse free survival. Notes: Hazard ratio for relapse, or death along the x-axis, and results from all trials on the y axis with gray squares representing effect estimates and lines through them representing 95% CIs. The gray diamond represents the overall effect measure, which lies clear off the line of no effect and shows a benefit for the treatment groups compared to placebo with a summary hazard ratio of 0.57 (95% CI: 0.45–0.71). The percentage weight for each study is separately listed on the right side of the graph as well as the data on the heterogeneity of the meta-analysis, with the relevant measure being the I^2^ score. CI, confidence interval.

Adjuvant treatment resulted consistently in longer RFS compared to placebo (HR 0.57; 95% CI= 0.45–0.7) (**Figure 2**). Patients in stage IV treated with nivolumab/ipilimumab derived the highest benefit (HR 0.23; 97.5% CI= 0.12–0.45). Pembrolizumab and nivolumab demonstrated similar efficacy, (HR 0.57; 95% CI= 0.43–0.74 and HR 0.56; 95% CI= 0.33–0.94 respectively). Patients with stage III BRAF*mut* MM treated with dabrafenib/trametinib had a 51% lower risk of relapse (HR 0.49; 95% CI= 0.40–0.59). Adjuvant therapy with ipilimumab was less effective (HR 0.76; 95% CI= 0.64–0.89). BRIM8 did not reach its primary endpoint in cohort 2 (stage IIIC MM, HR 0.81; 95%CI= 0.55–1.19). In cohort 1 however, treatment with vemurafenib resulted in longer RFS (stages IIC–IIIA, HR 0.55; 95% CI= 0.38–0.80).

### Subgroup Analyses

An overview of patient characteristics and demographics is provided in **eTables 2** and **3** in the **supplement**.

### Age

**Figure 3:**
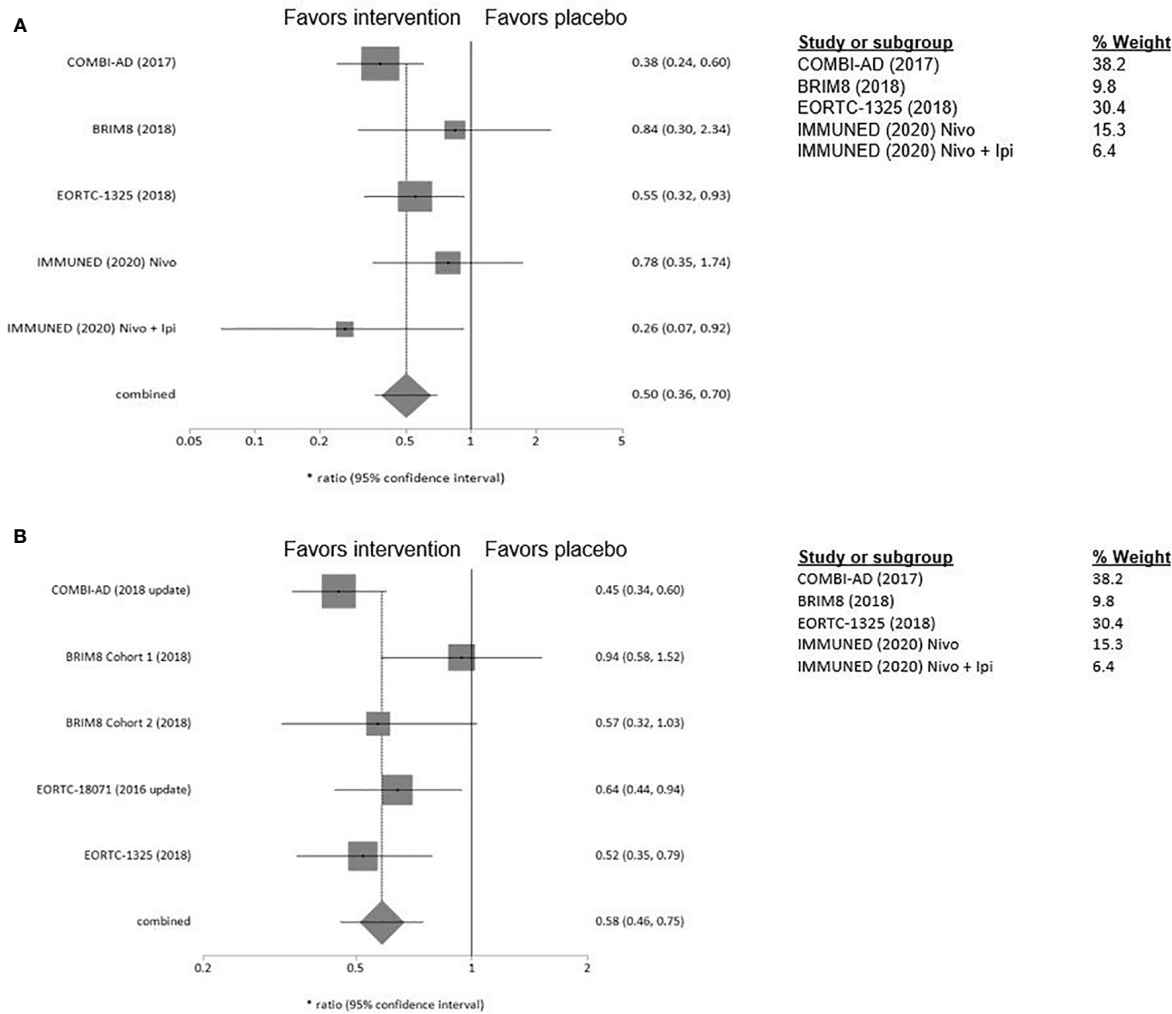
**(A)** Forest plot for primary outcome analysis on relapse free survival for patients **≥65 years old**. **(B)** Forest plot for primary outcome analysis on survival for patients <**65 years old**. Notes: Hazard ratio for relapse or death along the x-axis, and results from the different studies, with gray squares representing effect estimates and lines through them representing 95% CIs. The gray diamond represents the overall effect measure which lies clear off the line of no effect, showing a benefit for the treatment groups compared to placebo. The percentage weight for each study is separately listed on the right of the graph. CI, confidence interval.

No difference in adjuvant treatment benefit for patients aged over and under 65 years could be observed [≥65: HR 0.50 (95% CI= 0.36–0.70), <65: HR 0.58 (95% CI= 0.46–0.75)]. The greatest benefit of adjuvant therapy over placebo for patients ≥65 years was shown in the IMMUNED trial (HR 0.26; 95% CI= 0.07–0.92) (**Figure 3**).

### Lymph Node Involvement

Neither the number of involved LN, nor the presence of macro- or micrometastases alone had significant influence on RFS. (**eFigures 2** and **3, supplement**).

### Ulceration Status

**Figure 4:**
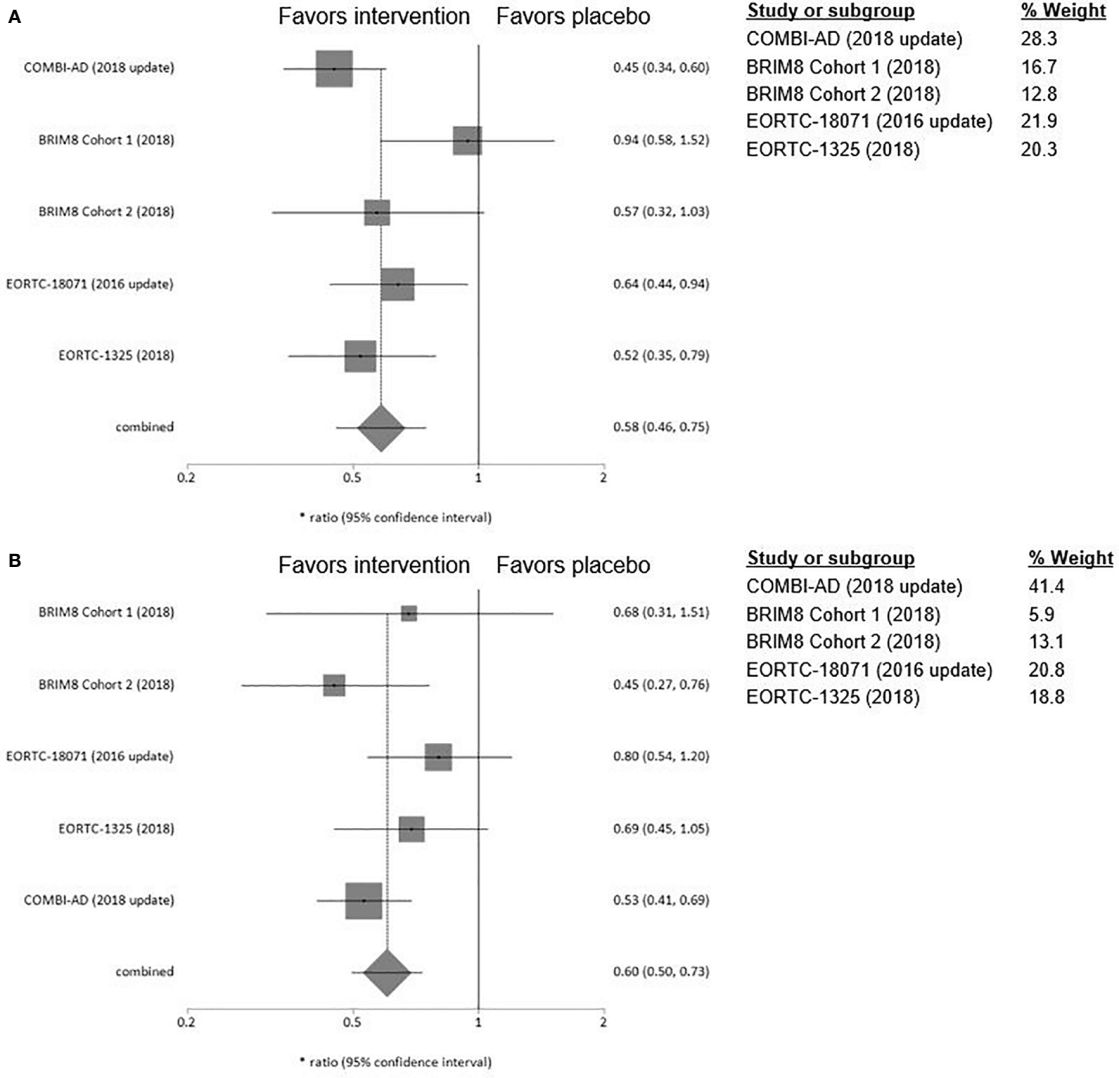
**(A)** Forest plot for primary outcome analysis on relapse free survival for patients **with ulcerated primary tumor**. **(B)** for patients **with non-ulcerated primary tumor.** Hazard ratio for relapse or death along the x-axis, and trial results on the y axis, with gray squares representing effect estimates and lines through them representing 95% CIs. The gray diamond represents the overall effect measure which lies clear of the line off no effect, showing a benefit for the treatment groups compared to placebo. The percentage weight for each study is separately listed on the right of the graph. CI, confidence interval.

In patients with ulcerated MM pembrolizumab and ipilimumab appeared to be more effective than in patients with non-ulcerated melanomas (pembrolizumab: HR 0.52; 95% CI 0.35–0.79 vs 0.68; 95% CI= 0.45–1.05, ipilimumab: HR 0.64, 95% CI= 0.44–0.94, vs 0.80, 95%CI= 0.54–1.20) (**Figure 4**). Interestingly, clinical benefit from dabrafenib/trametinib was consistent regardless of LN involvement or ulceration. Adjuvant therapy in non-ulcerated melanomas with macro-metastases was associated with the smallest RFS benefit and did not reach statistical significance (HR 0.73; 95%CI= 0.50–1.05) (**eFigure 4, supplement**).

### Stage

**Figure 5:**
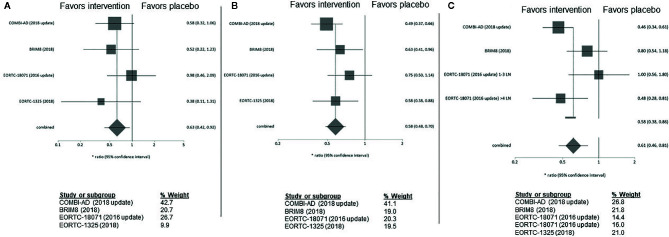
**(A)** Forest plot for primary outcome analysis on relapse free survival for patients with stage **IIIA**; **(B)** melanoma stage **IIIB** and **C** stage **IIIC** Hazard ratio for relapse or death along the x-axis, and results from all four studies; in **(C)** upper value for the EORTC18071 study includes patients in stage IIC with 1–3 LN, lower value with 4 LN, with gray squares representing effect estimates and lines through them representing 95% CIs. The gray diamond represents the overall effect measure which lies clear of the line off no effect, showing a benefit for the treatment groups compared to placebo. The percentage weight for each study is separately listed underneath the graph. CI, confidence interval; LN, lymph nodes.

In stage IIIA, while none of the examined substances alone reach statistical significance in the corresponding trials, our meta-analysis demonstrates a clear RFS-benefit for treatment versus placebo in stage IIIA, which in fact is numerically equivalent to that shown for stages IIIB/C. Dabrafenib/trametinib were associated with a consistent improvement in RFS, apart from stage IIIA where the upper confidence interval is marginally crossed (HR 0.58; 95% CI= 0.32–1.06). In contrast, ipilimumab had limited efficacy in patients with stage IIIA/B whereas a clear benefit with treatment was seen only in stage IIIC with >4 LN (HR 0.48; 95%CI= 0.28–0.81). Consistently, pembrolizumab also demonstrated a non-statistically significant benefit in stage IIIA (HR 0.38; 95%CI= 0.11–1.31) while higher stages (IIIB/C) clearly profit from adjuvant pembrolizumab treatment (**Figure 5**). The BRIM8 trial was the only to include patients with stage IIC. Here, median RFS was not reached in the vemurafenib arm.

### BRAF Mutation

The IMMUNED and EORTC-1325 trials reported separate outcomes as per BRAF mutational status. The presence of a BRAF mutation was associated with higher RFS rates (HR 0.30; 95% CI= 0.11–0.78) compared to the BRAF wildtype group (HR 0.60; 95% CI= 0.44–0.81). Nivolumab/ipilimumab was associated with the highest benefit in BRAF*mut* MM (HR 0.07; 95% CI= 0.02–0.23) (**eFigure 5, supplement**).

### Secondary Endpoints

Cross-trial comparison of secondary end points like OS and distant metastases free survival (DMFS) was not possible due to considerable variability in endpoint selection and reporting. In the EORTC-18071 trial, adjuvant therapy with ipilimumab significantly prolonged DMFS and most importantly OS (HR 0.72; 95.1% CI= 0.58–0.88) (20). In the COMBI-AD trial, data on OS were only reported for the first interim analysis. Treatment with BRAF/MEKi demonstrated higher 3-years OS-rates than with placebo (86% vs. 77% HR 0.57; 95%CI= 0.42–0.79) (21). EORTC-1325 demonstrated that pembrolizumab has maintained the health-related quality of life (22). The BRIM8 study demonstrated a DMFS of 37.2 months; in cohort 1, the DMFS was not reached.

### Adverse Events

The highest rate of grades 3–4 adverse events (AE) was observed with the nivolumab/ipilimumab combination (82%) with a treatment discontinuation rate of up to 62% (**eTable 4, supplement**). Ipilimumab monotherapy and vemurafenib were also associated with high grade 3-4 AE rates (54% and 59%, respectively) and discontinuation rates of 52% and 20%, respectively. Five deaths were attributed to ipilimumab monotherapy. 26% of patients treated with dabrafenib/trametinib went off study due to AE. One fatal serious AE (pneumonia) was reported in the combination-therapy group. In the EORTC-1325 trial, 13.8% of the patients discontinued pembrolizumab due to AE, which were equal to or higher than grade 3 in 31.6% of cases. There was one pembrolizumab related death due to myositis. Similar AE rates were observed with nivolumab monotherapy, with grades 3–4 toxicity up to 41% and 13% treatment discontinuation rate.

### Risk of Bias

The funnel plot (**eFigure 6, supplement**) and the result from Egger’s test (p = 0.311) showed indication of a publication bias. However, due to the limited number of studies included in the meta-analysis, this should be interpreted with caution. Overall, the trials were deemed to be at low risk for bias, except for “deviation of intended intervention” bias, for which it was unclear, whether participants with missing outcome data were excluded. In the COMBI-AD trial RFS was the prespecified outcome measurement however its estimation at 3 years was not prespecified (**eFigures 7** and **8, supplement**).

## Discussion

The RFS-benefit of modern adjuvant therapy (HR 0.57, 95% CI= 0.45–0.71) is higher than that shown for IFN-α in previous meta-analyses (HR 0.82, 95% CI = 0.77–0.87) (23).

Immune checkpoint inhibitor (ICi) monotherapy with pembrolizumab or nivolumab is similarly effective in improving RFS and both agents seem to be superior to ipilimumab while being less toxic, as previously demonstrated for nivolumab in the Checkmate-238 trial (24). This trial was not included in the current meta-analysis because of the lack of a placebo arm. An indirect analysis of adjuvant nivolumab versus placebo in stage III MM based on the Checkmate-238 and EORC-18071 trials calculated a HR for RFS of 0.53 (95% CI = 0.41–0.68) which is similar to the HR for RFS in the IMMUNED trial in stage IV (25).

The BRAF/MEKi combination therapy in the COMBI AD trial was associated with a clinical benefit across all subgroups with a tolerable adverse effect profile. In fact, the combination therapy demonstrated the highest numerical RFS-benefit in stage III melanoma (HR 0.49; 95% CI= 0.40–0.59). In stage IV melanoma with NED, nivolumab/ipilimumab demonstrated an impressive RFS benefit (HR 0.23; 97.5% CI= 0.12–0.45). The superiority of the combination versus ipilimumab or nivolumab monotherapy has also been demonstrated in metastatic melanoma, although at the cost of more grades 3–4 AE [24, 32]. However, only interim results from the rather small IMMUNED trial are currently published, and thus they must be interpreted with caution. Furthermore, stage IV patients with NED are per se at a higher risk of relapse, thus RFS benefits with adjuvant therapy between stages III and IV are not comparable.

In the interferon era, BRAF*mut* MM has been independently associated with a worse overall survival with HR of 1.7 (95% CI= 1.37–2.12) (26). Another, more recent meta-analysis of 52 trials also found that the presence of a BRAF mutation was associated with a reduced OS (HR 1.23, 95% CI= 1.09–1.38) (27). Most trials however included neither BRAF/MEKi nor ICi therapy. Prognosis of BRAF*mut* MM is expected to be crucially influenced by modern therapeutic agents. ICi have dramatically improved outcomes in the adjuvant and metastatic setting and additionally, patients with a BRAF mutation have now the option of targeted therapy. Thus, their prognosis can be expected to improve in the context of modern therapy. Interestingly, in our analysis, BRAF*mut* resectable MM was associated with higher RFS in trials which reported outcomes according to BRAF mutational status (IMMUNED and EORTC-1325). In contrast, in advanced melanoma, Puzanov and colleagues found in a pooled analysis of three RCTs with ICi (pembrolizumab) that BRAF*mut* patients had similar OS as patients with BRAF wild-type MM (PFS; 19.8% and 22.9% and OS; 35.1% and 37.5%). Patients with BRAF*mut* MM who did not receive BRAFi +/- MEKi therapy had a worse prognosis than those who did (28). This contradiction could be explained through the fact that, while BRAF mutations are early events in their evolution (29), metastatic melanomas accumulate further genomic alterations such as whole-genome duplication over time, which may account for resistance to treatment (30). Furthermore, the higher tumor burden of unresected melanomas might lead to increased potential of developing resistant clones under BRAF/MEKi compared to completely resected melanoma. These two factors could account for the discordance in the prognostic influence of BRAF mutations between completely resected stage III/IV in our meta-analysis and advanced/unresectable melanoma in the meta-analysis by Puzanov et al.

Age does not influence outcomes after adjuvant therapy. Specifically, the HR for RFS in patients ≥65 years old is even numerically lower than that of their younger counterparts. Therefore, advanced age alone should not discourage administration of adjuvant therapy. Recently published data on elderly patients with MM receiving ICi also demonstrated good clinical outcomes without increased toxicity (31).

Ulceration status of the primary tumor may be predictive of RFS when ICi are used. Several studies have shown that ulcerated melanomas have distinct biologic characteristics (32, 33). In our study, ipilimumab and pembrolizumab are both associated with a significant RFS benefit in patients with ulcerated melanoma, but not in those with primary tumors without ulceration. In contrast, dabrafenib/trametinib showed benefit regardless of ulceration status, while vemurafenib is also associated with superior RFS in non-ulcerated stage IIIC MM. A *post hoc* meta-analysis of the EORTC-18952 (IFN α-2b versus observation in stages IIB–III) and -18991 trials (pegylated-IFN versus observation in stage III) also demonstrated that the absence of ulceration was predictive for inefficacy of adjuvant treatment with IFN-α (34). Therefore, it would be reasonable to prefer a BRAF/MEKi combination in non-ulcerated BRAF*mut* MM.

Adjuvant treatment in stage IIIA is associated with a similar RFS-benefit as in stages IIIB/C. Thus, our meta-analysis supports administration of adjuvant therapy in stage IIIA. However, all the above trials are powered for DFS/RFS and OS data have only been reported for the first interim analysis of the COMBI-AD trial and for the EORC-18071 trial, where a benefit could be demonstrated in favor of treatment. The recently reported update of the Checkmate-238 trial (35) showed no difference in OS between ipilimumab and nivolumab despite a significant RFS benefit. This and the general lack of OS data pose the issue of early vs late treatment, particularly in stages II/IIIA. Further data on OS are needed to guide treatment decisions. The influence of toxicity in decision making in earlier stages is discussed below.

As IFN-α has been associated with substantial AE and drug related fatalities, modern adjuvant therapeutics have to meet high expectations (23, 36, 37). The highest toxicity was seen with the nivolumab/ipilimumab combination in stage IV MM with NED. Pembrolizumab toxicity in the adjuvant setting (31.6% grade ≥3 AE) was lower compared to the AE rate of nivolumab (41%). However, data on nivolumab toxicity in our meta-analysis are derived from stage IV MM, while pembrolizumab was tested in stage III patients. The Checkmate238 trial, which also included stage III patients demonstrated a 25.4% rate of grades 3–4 AE in patients treated with nivolumab (11). Monotherapy with pembrolizumab or nivolumab have been shown to have a considerably better tolerability profile than ipilimumab (10, 24, 38). The dabrafenib/trametinib combination demonstrated similar grades 3–4 AE rates as pembrolizumab and nivolumab (26%) and less than half compared to vemurafenib (59%), as in previous trials comparing BRAF/MEKi combinations to BRAFi monotherapy (39, 40). The ICi combination and ipilimumab monotherapy were associated with the highest toxicity. In the context of the curative adjuvant setting, potentially permanent toxicities involved with ICi become particularly relevant. The incidence is higher with ICi combination than with PD-1 monotherapy (hypophysitis: 8.0% vs. 1.1, hypothyroidism: 13.2% vs. 7.0%). Primary adrenal insufficiency and insulin dependent diabetes are rare events (cumulative incidence after ICi: 0.7% and 0.2% respectively) (41–43). Because of this potentially long-lasting toxicity and the lack of consistent data on OS as discussed above we generally prefer BRAF/MEKi as adjuvant treatment in the context of BRAF-mutant MM, especially in stages IIIA/B.

An important unanswered clinical question is adjuvant therapy for patients with stage II MM, where rates of distant recurrence after resection can reach 44% (44, 45). Vemurafenib monotherapy is not approved in the adjuvant therapy of MM, however, BRIM8 was the only trial to include patients with stage IIC. In this subgroup, no events occurred in the vemurafenib arm (0/15) whereas six patients suffered a relapse in the placebo arm (6/12) (13). Although IFN-α remains an adjuvant therapeutic option for patients with stages IIB and IIC melanoma, it is rarely used in daily practice due to its significant toxicity (9). Currently ongoing trials are comparing pembrolizumab and nivolumab vs. placebo in resected stage II MM (45, 46).

Neoadjuvant approaches with both BRAF/MEKi and ICi in high-risk resectable MM are also currently under investigation (47, 48).

Although based on well-designed trials with robust results, our meta-analysis still has some limitations. First, it does not address the contemporary question of a comparison between adjuvant and neoadjuvant therapy. Second, it was restricted to placebo-controlled studies and thus forced the exclusion of relevant trials like those comparing two agents (e.g., ipilimumab versus nivolumab). Moreover, due to the significant trial variability regarding endpoint reporting and lacking consistent OS data reporting/availability, our meta-analysis is based on RFS and not on OS data. On this matter, significant inter-trial heterogeneity is also noted in the stages included. Furthermore, comparisons between subgroups were not possible for all the included RCTs, as subgroup-definition as well as data availability for each subgroup varied across the trials. Another limitation is the inconsistent representation of BRAF*mut* MM across trials. In addition, treatment and definition of stage III within the included RCTs does not correspond completely to current standards. Moreover, complete lymph node dissection was required for trial enrollment in the EORTC-1325, EORTC-18071 and COMBI-AD trials. This practice has been meanwhile replaced by sentinel lymph node biopsy according to results from RCTs (49, 50). Additionally, it must be kept in mind, that the current definition of stage III disease according to the 8^th^ edition of AJCC is different than the one used in the RCTs above. Stages IIIA/B/C as defined in the 7^th^ edition carry a worse prognosis, and may therefore benefit more from adjuvant therapy (51).

In conclusion, contemporary adjuvant therapy in the post interferon-alpha era for patients with high-risk completely resected MM is effective and tolerable and should be recommended in all patients in the absence of contraindications. BRAF*mut* MM was associated with higher RFS. Furthermore, some subgroups may benefit more from specific treatments and this can guide treatment choice. Advanced age and stage IIIA should not discourage adjuvant treatment. Options in BRAF wildtype melanoma are limited to ICi. In BRAF*mut* MM, BRAF/MEKi should be preferred, especially in the absence of ulceration and stage IIIA. Adjuvant treatment should be adapted to patient preference like the intake schedule or pre-existing conditions.

## Data Availability Statement

The data sets presented in this study can be found in online repositories. The names of the repository/repositories and accession number(s) can be found below: “The Open Science Framework—Center for OpenScience” (https://osf.io/m9vr5).

## Author Contributions

KC, KY, and DK-M performed the systematic review. KC, KY, MB, LT, and TR performed the data analysis. KC, CP, CM, TV, SS, and DK-M evaluated the data. KC, KY, and DK-M drafted the manuscript. All authors contributed to the article and approved the submitted version.

## Conflict of Interest

The authors declare that the research was conducted in the absence of any commercial or financial relationships that could be construed as a potential conflict of interest.
