# Supplementary material for: Adjuvant Therapy of High-Risk (Stages IIC–IV) Malignant Melanoma in the Post Interferon-Alpha Era: A Systematic Review and Meta-Analysis
Source: Front Oncol. 2021 Feb 18;10:637161. doi: 10.3389/fonc.2020.637161 (PMC7930562; doi:10.3389/fonc.2020.637161)
Supplement: Supplementary file 1 [file DataSheet_1.docx]

Supplement

**Figures:**


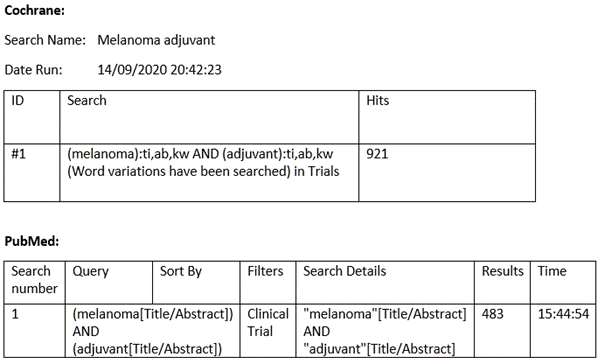


**eFigure 1.** Search Strategy


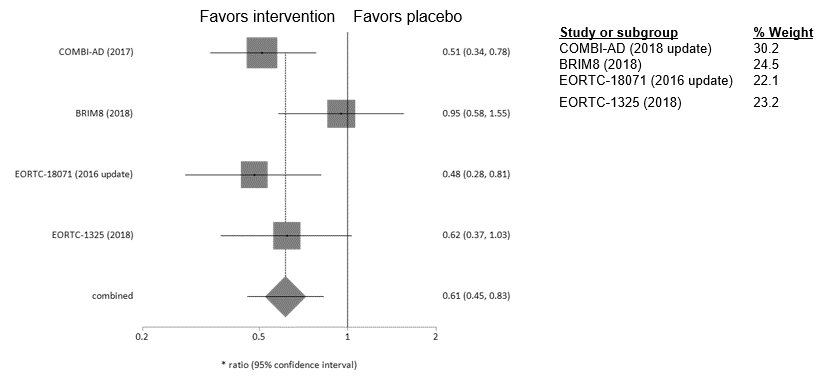

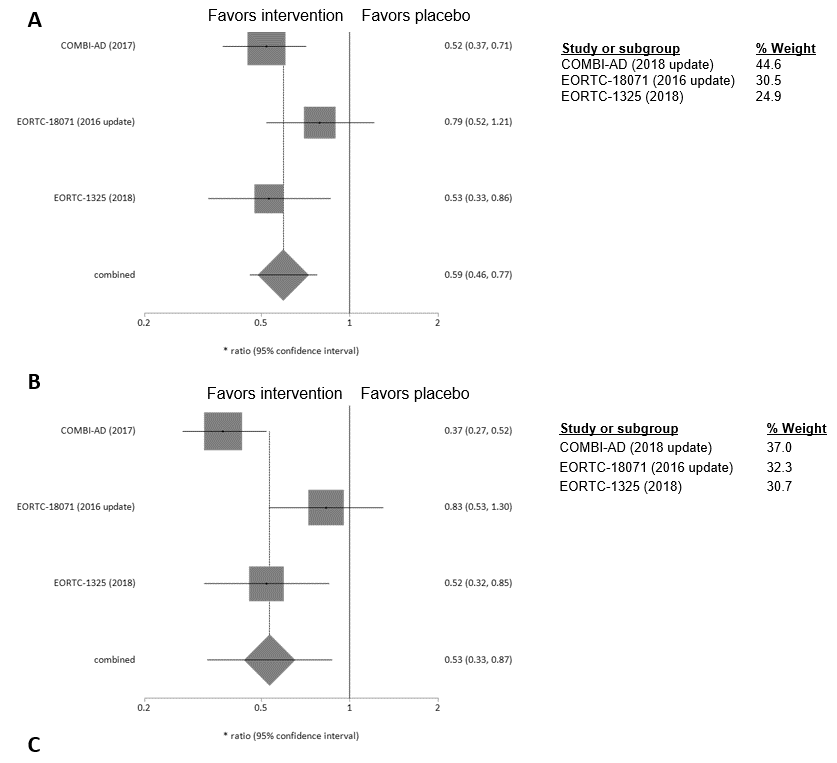


**eFigure 2. A:** Forest plot for primary outcome analysis on relapse free survival for patients with 1 LN. **B:** Forest plot for primary outcome analysis on survival for patients with 2-3 LN. **C:** Forest plot for primary outcome analysis on survival for patients with 4 LN Notes: Hazard ratio for relapse or death along the x-axis, and trial results on the y axis, with gray squares representing effect estimates and lines through them representing 95% CIs. The gray diamond represents the overall effect measure which lies clear of the line off no effect. The percentage weight for each study is separately listed on the right of the graph. Abbreviations: CI, confidence interval. LN: lymph node


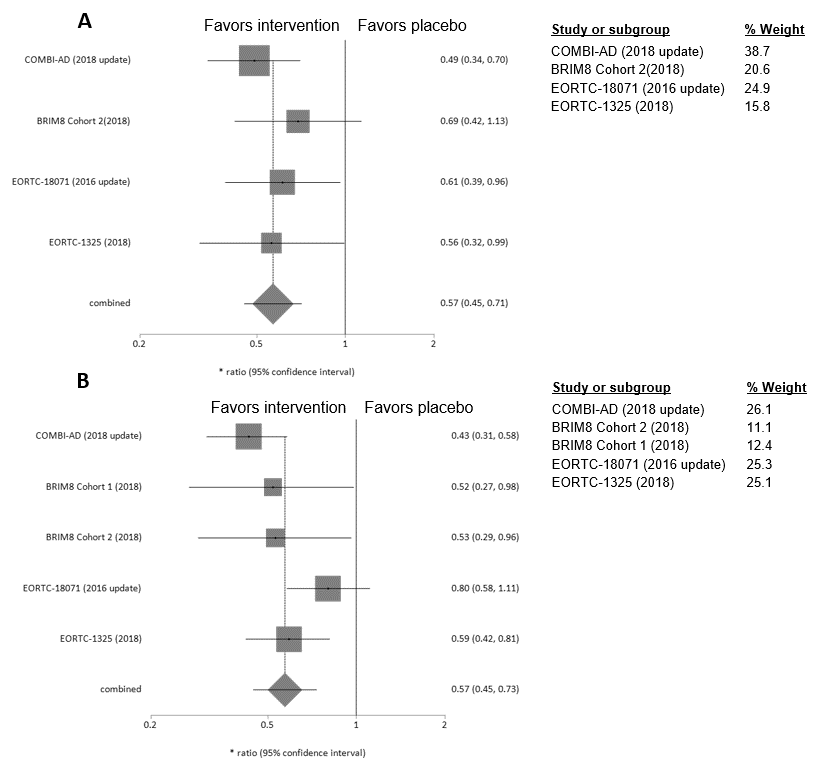


**eFigure 3. A:** Forest plot for primary outcome analysis on relapse free survival for patients with micrometastases. **B:** and for patients with macromestasis. Hazard ratio for relapse or death along the x-axis, and trial results on the y axis, with gray squares representing effect estimates and lines through them representing 95% CIs. The gray diamond represents the overall effect measure which lies clear off the line of no effect, showing a benefit for the treatment groups compared to placebo. The percentage weight for each study is separately listed on the right of the graph. Abbreviations: CI, confidence interval.


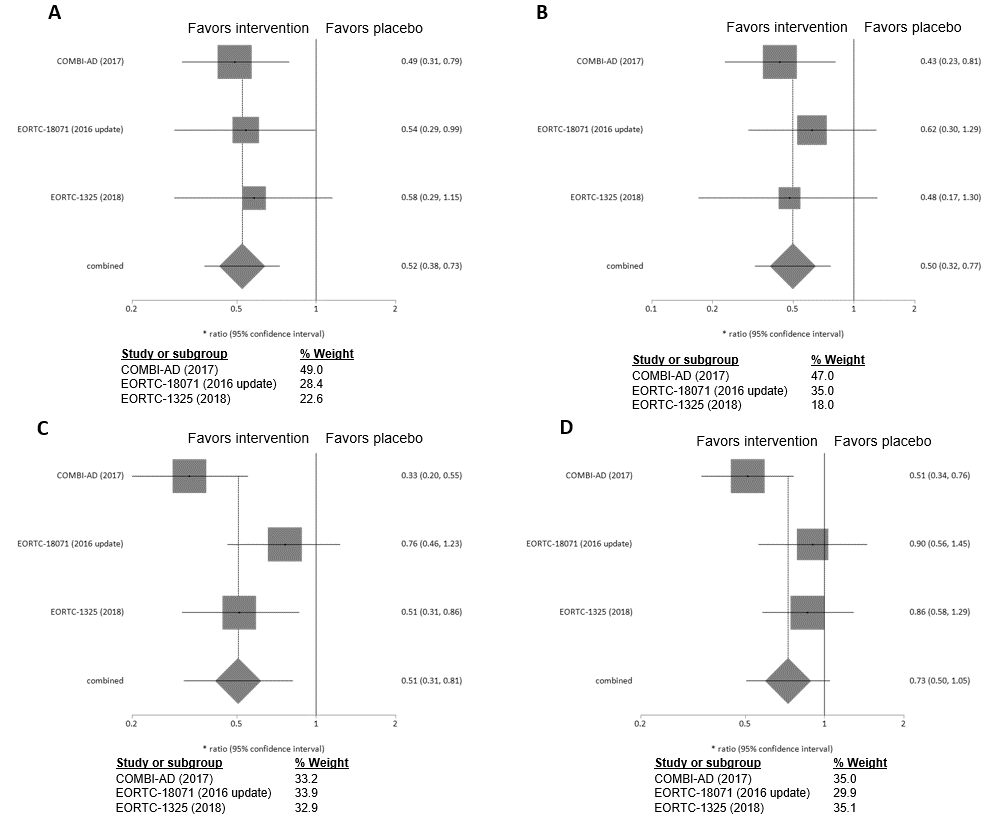


**eFigure 4. A:** Forest plot for primary outcome analysis on relapse free survival for patients with **micrometastases and ulcerated primary tumor**. **B:** patients with **micrometastases and no ulcerations**; **C:** with **macrometastases with ulcerated primary tumor**; **D:** **macrometastases and no ulcerations**. Hazard ratio for relapse or death along the x-axis, and trial results on the y axis, with gray squares representing effect estimates and lines through them representing 95% CIs. The gray diamond represents the overall effect measure which lies clear off the line of no effect, showing a benefit for the treatment groups compared to placebo. The percentage weight for each study is separately listed on the right of the graph. Abbreviations: CI, confidence interval.


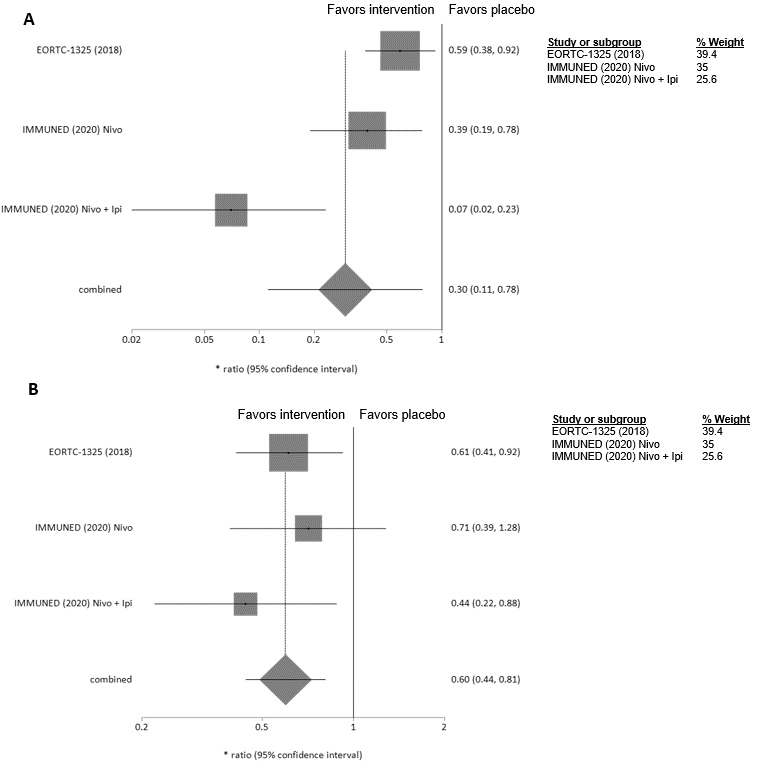


**eFigure 5.** **A:** Forest plot for primary outcome analysis on relapse free survival for patients with BRAF mutation; **B:** patients with BRAF wildtype melanoma. Hazard ratio for relapse or death along the x-axis, and trial results on the y axis, with gray squares representing effect estimates and lines through them representing 95% CIs. The gray diamond represents the overall effect measure which lies clear of the line off no effect, showing a benefit for the treatment groups compared to placebo. The percentage weight for each study is separately listed on the right of the graph. Abbreviations: CI, confidence interval.


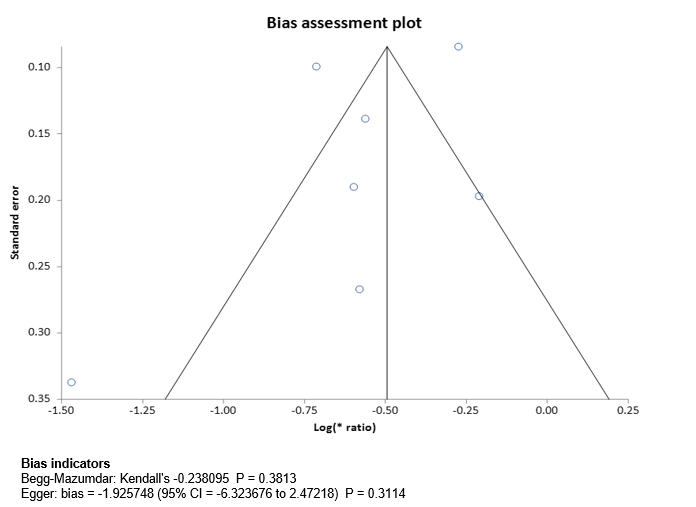


**eFigure 6**. Funnel plot for publication bias for primary outcome analysis on survival

**eFigure 7: Risk of bias graph.** Authors' judgements about each risk of bias category presented as percentages across all included trials.


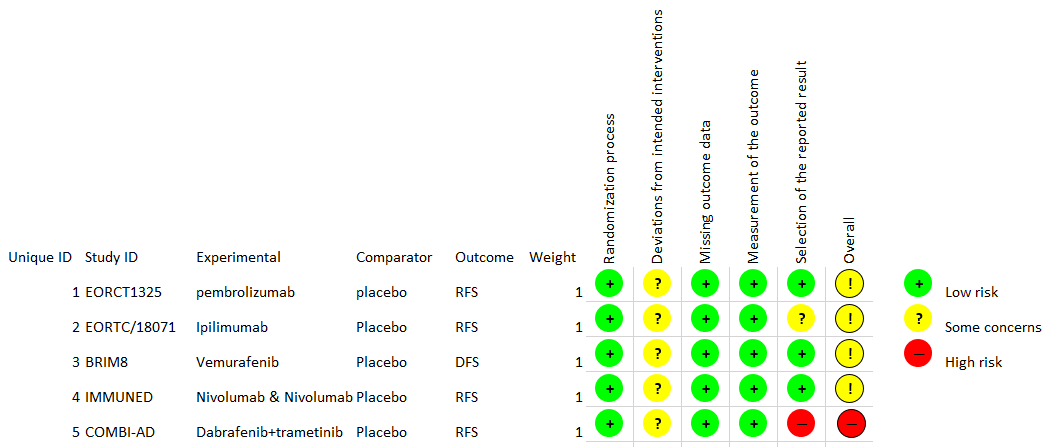


**eFigure 8: Risk of bias graph.** Risk of bias presented in colour scale

| **Section/topic** | **#** | **Checklist item** | **Reported on page #** |
| --- | --- | --- | --- |
| **TITLE** | | |  |
| Title | 1 | Identify the report as a systematic review, meta-analysis, or both. | 1 |
| **ABSTRACT** | | |  |
| Structured summary | 2 | Provide a structured summary including, as applicable: background; objectives; data sources; study eligibility criteria, participants, and interventions; study appraisal and synthesis methods; results; limitations; conclusions and implications of key findings; systematic review registration number. | 2 |
| **INTRODUCTION** | | |  |
| Rationale | 3 | Describe the rationale for the review in the context of what is already known. | 3 |
| Objectives | 4 | Provide an explicit statement of questions being addressed with reference to participants, interventions, comparisons, outcomes, and study design (PICOS). | 3 |
| **METHODS** | | |  |
| Protocol and registration | 5 | Indicate if a review protocol exists, if and where it can be accessed (e.g., Web address), and, if available, provide registration information including registration number. | 4 |
| Eligibility criteria | 6 | Specify study characteristics (e.g., PICOS, length of follow-up) and report characteristics (e.g., years considered, language, publication status) used as criteria for eligibility, giving rationale. | 4 |
| Information sources | 7 | Describe all information sources (e.g., databases with dates of coverage, contact with study authors to identify additional studies) in the search and date last searched. | 4 |
| Search | 8 | Present full electronic search strategy for at least one database, including any limits used, such that it could be repeated. | 4 |
| Study selection | 9 | State the process for selecting studies (i.e., screening, eligibility, included in systematic review, and, if applicable, included in the meta-analysis). | 4 |
| Data collection process | 10 | Describe method of data extraction from reports (e.g., piloted forms, independently, in duplicate) and any processes for obtaining and confirming data from investigators. | 4 |
| Data items | 11 | List and define all variables for which data were sought (e.g., PICOS, funding sources) and any assumptions and simplifications made. | 4 |
| Risk of bias in individual studies | 12 | Describe methods used for assessing risk of bias of individual studies (including specification of whether this was done at the study or outcome level), and how this information is to be used in any data synthesis. | 4 |
| Summary measures | 13 | State the principal summary measures (e.g., risk ratio, difference in means). | 4 |
| Synthesis of results | 14 | Describe the methods of handling data and combining results of studies, if done, including measures of consistency (e.g., I^2^) for each meta-analysis. | 4 |

**Tables:**

| **Section/topic** | **#** | **Checklist item** | **Reported on page #** |
| --- | --- | --- | --- |
| Risk of bias across studies | 15 | Specify any assessment of risk of bias that may affect the cumulative evidence (e.g., publication bias, selective reporting within studies). | 4 |
| Additional analyses | 16 | Describe methods of additional analyses (e.g., sensitivity or subgroup analyses, meta-regression), if done, indicating which were pre-specified. | 4 |
| **RESULTS** | | |  |
| Study selection | 17 | Give numbers of studies screened, assessed for eligibility, and included in the review, with reasons for exclusions at each stage, ideally with a flow diagram. | 5 |
| Study characteristics | 18 | For each study, present characteristics for which data were extracted (e.g., study size, PICOS, follow-up period) and provide the citations. | 6 |
| Risk of bias within studies | 19 | Present data on risk of bias of each study and, if available, any outcome level assessment (see item 12). | 8 |
| Results of individual studies | 20 | For all outcomes considered (benefits or harms), present, for each study: (a) simple summary data for each intervention group (b) effect estimates and confidence intervals, ideally with a forest plot. | 6 |
| Synthesis of results | 21 | Present results of each meta-analysis done, including confidence intervals and measures of consistency. | 6-8 |
| Risk of bias across studies | 22 | Present results of any assessment of risk of bias across studies (see Item 15). | 8 |
| Additional analysis | 23 | Give results of additional analyses, if done (e.g., sensitivity or subgroup analyses, meta-regression [see Item 16]). | 7-8 |
| **DISCUSSION** | | |  |
| Summary of evidence | 24 | Summarize the main findings including the strength of evidence for each main outcome; consider their relevance to key groups (e.g., healthcare providers, users, and policy makers). | 8-11 |
| Limitations | 25 | Discuss limitations at study and outcome level (e.g., risk of bias), and at review-level (e.g., incomplete retrieval of identified research, reporting bias). | 11 |
| Conclusions | 26 | Provide a general interpretation of the results in the context of other evidence, and implications for future research. | 11 |
| **FUNDING** | | |  |
| Funding | 27 | Describe sources of funding for the systematic review and other support (e.g., supply of data); role of funders for the systematic review. | NA |

**eTable 1.** PRISMA Checklist

| **Trial** | **Arm** | **BRAF mutation (per arm)** | **BRAF mutation**  **(total)** | **N. of positive lymph nodes** | | | **Type of lymph node involvment** | | **Micrometastases and ulceration** | | **Macrometastases and ulceration** | |
| --- | --- | --- | --- | --- | --- | --- | --- | --- | --- | --- | --- | --- |
|  |  |  |  | 1 | 2-3 | >4 | Microscopic | Macroscopic | yes | no | yes | no |
| **EORC-18071** | Ipilimumab | - | - | 271 (46%) | 163 (34%) | 95 (20%) | 210 (44%) | 265 (56%) | 99 (47%) | 98 (46%) | 104 (39%) | 153 (57.7) |
|  | Placebo | - |  | 220 (46%) | 158 (33%) | 98 (21%) | 193 (41%) | 283 (59%) | 88 (45.5%) | 115 (59.5%) | 97 (34.2%) | 147 (51.9) |
| **COMBI-AD** | Dabrafenib + Trametinib | 438 (100%) | 870 (100%) | 177 (40%) | 158 (36%) | 73 (17%) | 152 (35%) | 158 (36%) | 64 (42%) | 87 (57.2%) | 58 (36.7%) | 100 (63.2) |
|  | Placebo | 432 (100%) |  | 183 (42%) | 150 (35%) | 72 (17%) | 157 (36%) | 161 (37%) | 79 | 78 | 58 | 101 |
| **BRIM8**  Cohort I | Vemurafenib | 157 (100%) | 310 (100%) | - | - | - | 84 (59%) | 58 (41%) | - | - | - | - |
|  | Placebo | 157 (100%) |  | - | - | - | 76 (52%) | 69 (48%) |  | - | - | - |
| **BRIM8**  Cohort II | Vemurafenib | 93 (100%) | 184 (100%) | - | - | 63 (68%) | - | 30 (32%) | - | - | - | - |
|  | Placebo | 91 (100%) |  | - | - | 64 (70%) | - | 27 (39%) | - | - | - | - |
| **EORTC-1325** | Pembrolizumab | 245 (47.7%) | 507 (49%) | 227 (44%) | 177 (34%) | 110 (21.4%) | 187 (36.4%) | 327 (63.6%) | 94 (44.7) | 89 (42.3) | 114 (43%) | 141 (53%) |
|  | Placebo | 262 (51.8%) |  | 237 (46.9%) | 166 (32.9%) | 102 (20.2%) | 161 (31.9%) | 344 (68.1%) | 75 | 85 | 122 | 166 |
| **IMMUNED** | Nivolumab + Ipilimumab | 27 (48%) | 75 (45%) | - | - | - | - | - | - | - | - | - |
|  | Nivolumab | 27 (46%) |  | - | - | - | - | - | - | - | - | - |
|  | Placebo | 21 (40%) |  | - | - | - | - | - | - | - | - | - |

**eTable 2**. Demographics and characteristics of patients at baseline, * all data refer to the study population

**eTable 3**. Demographics and characteristics of patients at baseline, * all data refer to the study population

**eTable 3**. Demographics and characteristics of patients at baseline, * all data refer to the study population

| **Trial** | **Substance** | **Patients enrolled* (n)** | **Gender** | | **Age** | | **Stage** | | | | | **Tumour ulceration** | |
| --- | --- | --- | --- | --- | --- | --- | --- | --- | --- | --- | --- | --- | --- |
|  |  |  | male | female | <65 | >65 | IIC | IIIA | IIIB | IIIC | IV | yes | no |
| **IMMUNED** | Nivolumab + Ipilimumab | 56 | 31 (55%) | 25 (45%) | 45 (80%) | 11 (20%) | - | - | - | - | 56 | - | - |
|  | Nivolumab | 59 | 31 (53%) | 28 (47%) | 43 (73%) | 16 (27%) | - | - | - | - | 59 | - | - |
|  | Placebo | 52 | 33 (63%) | 19 (37%) | 35 (67%) | 17 (33%) | - | - | - | - | 52 | - | - |
| **EORC-18071** | Ipilimumab | 475 | 296 (62%) | 179 (38%) | 394(83%) | 81 (17%) | - | 98 (21%) | 213 (38%) | 164 (41%) | - | 197 (41%) | 257 (54%) |
|  | Placebo | 476 | 293 (62%) | 183 (38%) | 389 (81%) | 87 (18%) | - | 98 (21%) | 182 (38%) | 196 (41%) | - | 293 (43%) | 244 (51%) |
| **COMBI-AD** | Dabrafenib + Trametinib | 438 | 195 (45%) | 243 (55%) | 353 (80%) | 85 (19.4) | - | 83 (19%) | 169 (39%) | 181 (41%) | - | 179 (41%) | 253 (58%) |
|  | Placebo | 432 | 193 (45%) | 239 (55%) | 359 (83.1%) | 73 (16.8) | - | 71 (16%) | 187 (43%) | 166 (38%) | - | 177 (41%) | 249 (58%) |
| **BRIM8**  **Cohort I** | Vemurafenib | 157 | 84 (54%) | 73 (46%) | 136 (87%) | 21 (13%) | 15 (10%) | 36 (23%) | 106 (68%) | - | - | 63 (44%) | 81 (56%) |
|  | Placebo | 157 | 88 (56%) | 69 (44%) | 137 (87%) | 20 (13%) | 12 (8%) | 39 (25%) | 106 (68%) | - | - | 49 (34%) | 94 (65.7%) |
| **BRIM8**  **Cohort II** | Vemurafenib | 93 | 52 (56%) | 41 (44%) | 77 (83%) | 16 (17%) | - | - | - | 93 (100%) | - | 57 (67%) | 28 (32%) |
|  | Placebo | 91 | 59 (65%) | 32 (35%) | 80 (88%) | 11 (12%) | - | - | - | 91 (100%) | - | 49 (63%) | 29 (37.1%) |
| **EORTC-1325** | Pembrolizumab | 514 | 324 (63%) | 190 (37%) | 389 (75.6%) | 125 (24.3%) | - | 80 (15.6%) | 237 (46.1%) | 197 (38.3%) | - | 208 (40.4%) | 230 (45%) |
|  | Placebo | 505 | 304 (60.2%) | 201 (39.8%) | 379 (75%) | 126 (25%) | - | 80 (15.8%) | 230 (45.5%) | 195 (38.6%) | - | 197 (39%) | 251 (49.7%) |

**eTable 3,** Demographics and characteristics of patients at baseline, * all of the data refer to the study population

at baseline, * all data refer to the study population

| **Trial** | **Substance** | **Evaluated patients** | **Adverse events** | **Grade I-II** | **Grade III-IV** | **Patients discontinuing treatment** | **Deaths** |
| --- | --- | --- | --- | --- | --- | --- | --- |
| **IMMUNED** | Nivolumab + Ipilimumab | 55 | 55 (100%) | - | 45 (82%) | 34 (62%) | 1 (not drug related) |
|  | Nivolumab | 56 | 54 (96%) | - | 23 (41%) | 7 (13%) | 2 (not drug related) |
|  | Placebo | 51 | 49 (96%) | - | 13 (25%) | 1 (2%) | 0 |
| **EORC-18071** | Ipilimumab | 471 | 465 (98%) | 205 (44%) | 254 (54%) | 245 (52%) | 6 (1%) (5 drug-related) |
|  | Placebo | 474 | 432 (91%) | 307 (65%) | 124 (26%) | 20 (4%) | 6 (1%) (0 drug-related) |
| **COMBI-AD** | Dabrafenib + Trametinib | 435 | 422 (97%) | - | 180 (41%) | 114 (26%) | 1 (drug related) |
|  | Placebo | 432 | 380 (88%) | - | 61 (14%) | 12 (3%) | 0 |
| **BRIM8** | Vemurafenib | 247 | 245 (99%) | 104 (42%) | 141 (59%) | 49 (20%) | 1 (not drug related) |
|  | Placebo | 247 | 219 (89%) | 182 (74%) | 37 (15%) | 5 (2%) | 0 |
| **EORTC-1325** | Pembrolizumab | 509 | 475 (93.3%) | - | 161 (31.6) *  (14.7% treatment related) | 70 (13.8%) | 3 (1 treatment related (myositis) |
|  | Placebo | 502 | 453 (90.2%) | - | 93 (18.5%) *  (3.4% treatment related) | 11 (2.2%) | 0 |

**eTable 4,** Adverse events characteristics * The EORTC-1325 trial reported grouped AE of grade III or higher
